# Supplementary material for: POD1-SUN-CRT3 chaperone complex guards the ER sorting of LRR receptor kinases in Arabidopsis
Source: Nat Commun. 2022 May 16;13:2703. doi: 10.1038/s41467-022-30179-w (PMC9110389; doi:10.1038/s41467-022-30179-w)
Supplement: Supplementary file 1 — Supplementary Information [file 41467_2022_30179_MOESM1_ESM.pdf]

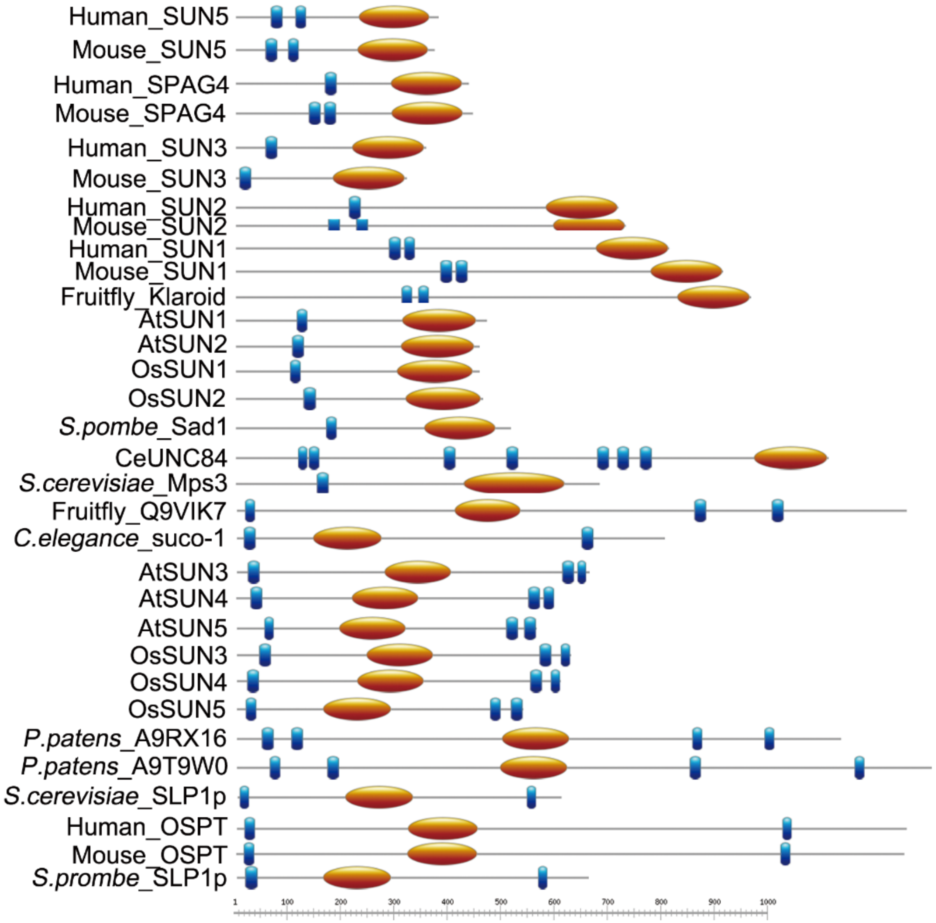

Supplementary Figure 1. Domain structure of SUN proteins in different organisms.

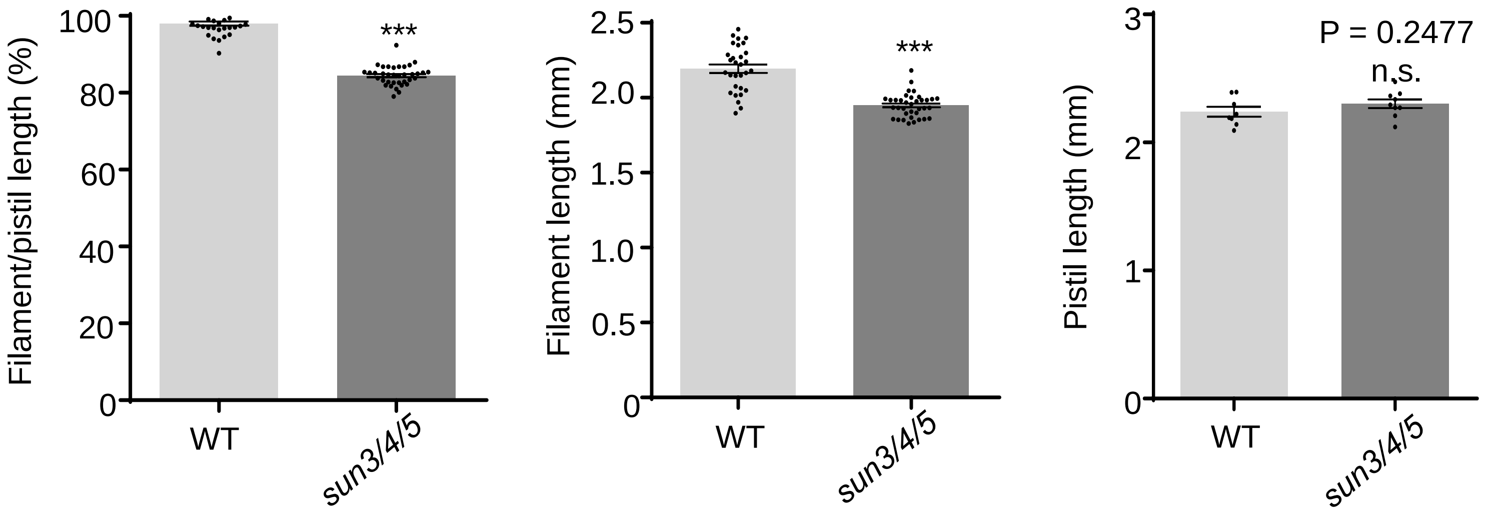

Supplementary Figure 2. Statistics of stamen filaments and pistils in WT and *sun3/4/5*.

The newly opened flowers were dissected and photographed with a stereo microscope. The length of the pistil and stamens was measured. The values represent means ± s.e.m., Two-tailed Students *t*-test, p*** < 0.001. 86 WT and 107 *sun3/4/5* filaments were measured. 23 WT and 27 *sun3/4/5* pistils were measured. For filament/pistil length ratio, n = 90 for WT and n = 106 for *sun3/4/5.* Each dot in the bar graph represents the length of each longer stamen filament (4 in each flower).

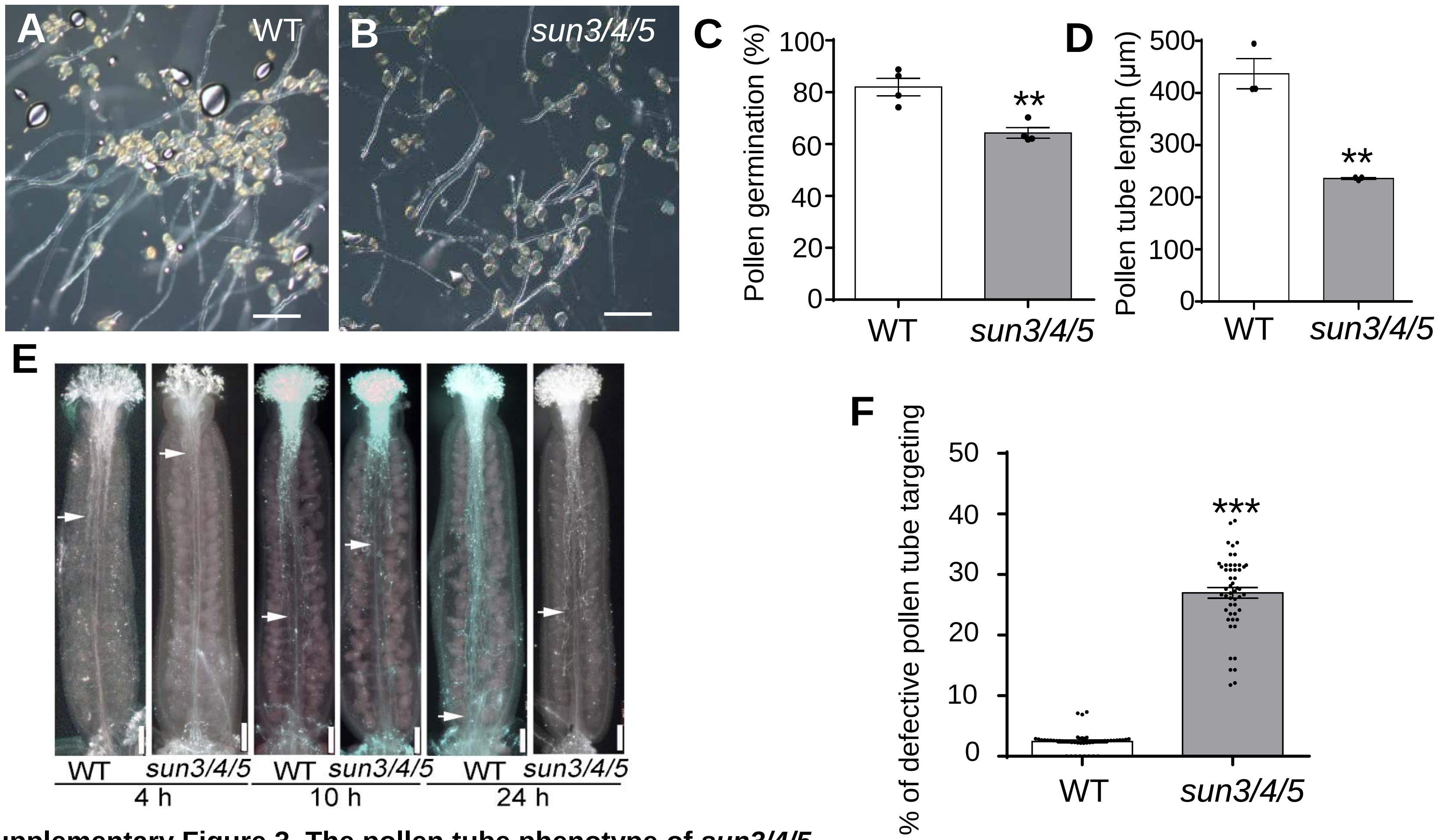

Supplementary Figure 3. The pollen tube phenotype of *sun3/4/5*.

A and **B.** *In vitro* pollen germination and tube growth of WT and *sun3/4/5*. Bar, 100 μm. **C** and **D**, Statistics of pollen germination ratio and pollen tube length. The values represent means ± s.e.m., Two-tailed Student's *t*-test, p** = 0.004 , 4 independent experiments were conducted in **C**. p** = 0.0023, 323 WT and 380 *sun3/4/5* pollen tubes were counted in **D**. **E**. *sun3/4/5* shows defective pollen tube growth *in vivo*. The arrows point where the longest pollen tube reached in each pistil. Each panel is representative of six pistils. h, hours after pollination. Scale bar, 200 μm. **F**. Quantification of ovule targeting ratio. The values represent means ± s.e.m., Two-tailed Student's *t*-test, p*** < 0.001, n = 48 and n = 51, respectively, for WT and *sun3/4/5*.

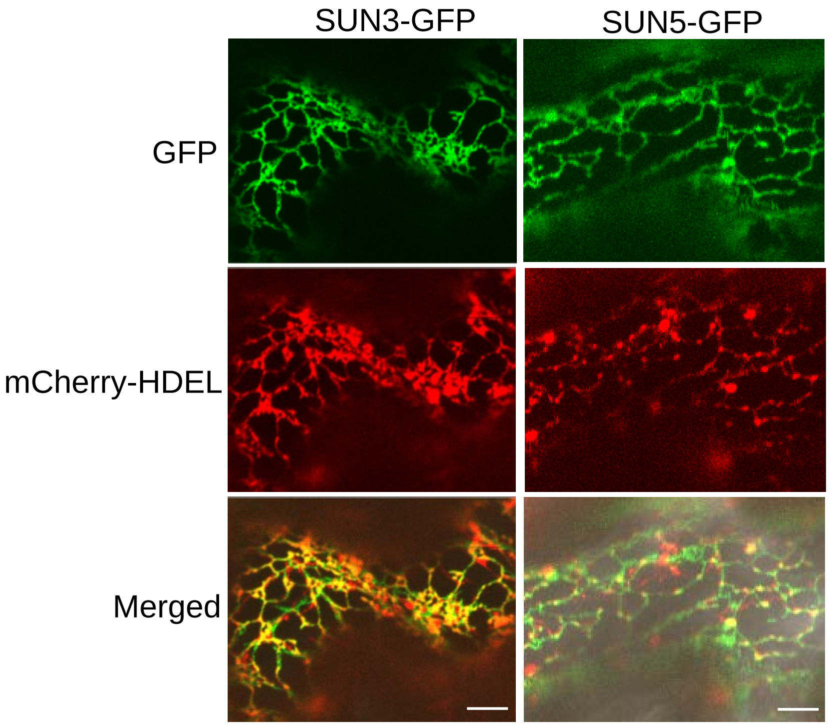

Supplementary Figure 4. Subcellular localization of SUN3 and SUN5.

Co-expression of SUN3-GFP and SUN5-GFP driven by *35S* promoter, with the ER marker mCherry-HDEL, respectively, in tobacco leaves. 3 independent biological experiments were repeated. Bar, 10 μm.

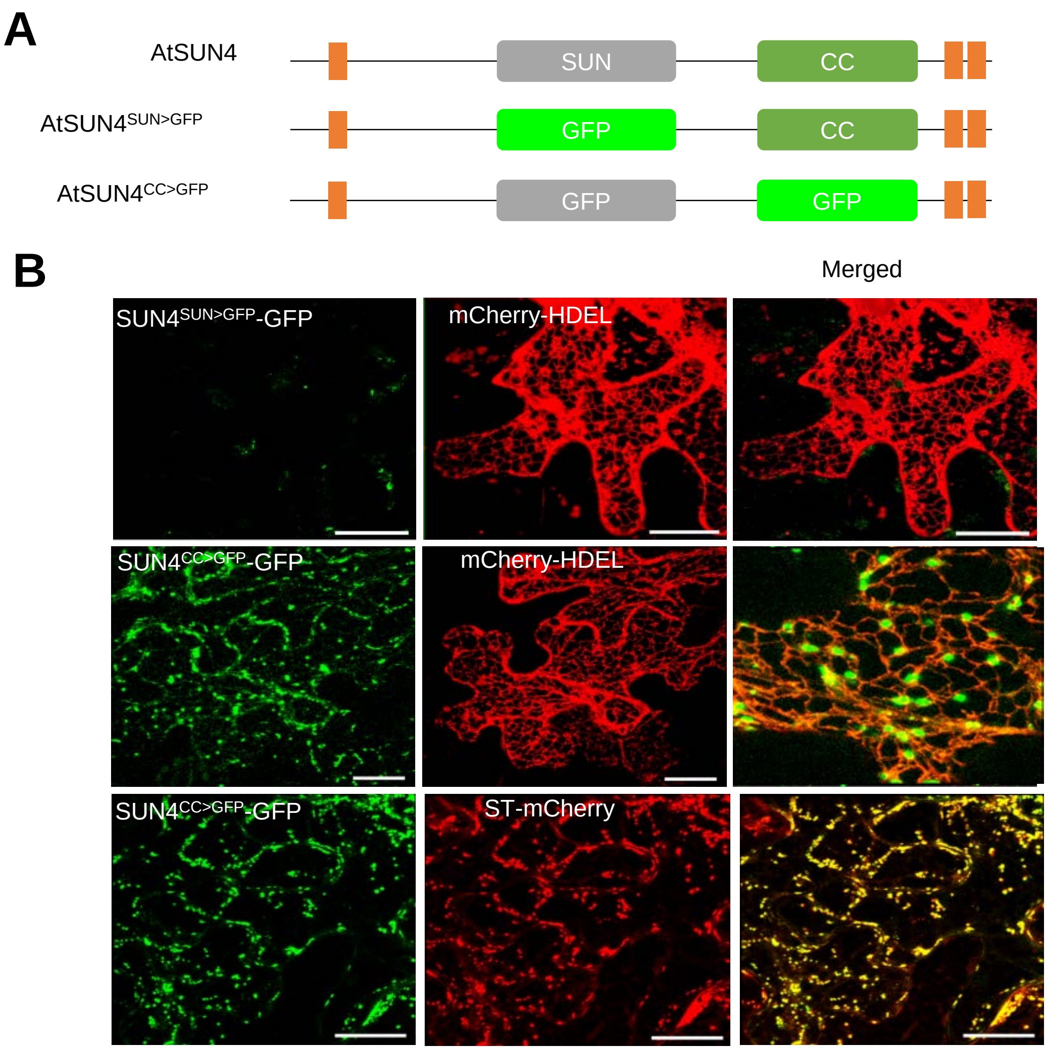

Supplementary Figure 5. Functional analysis of different domains of SUN4.

A. Diagram of the chimeric proteins of SUN4. **B**. The subcellular localization of SUN4 mutations in tobacco. 3 independent biological experiments were repeated. Bar, 10 μm.

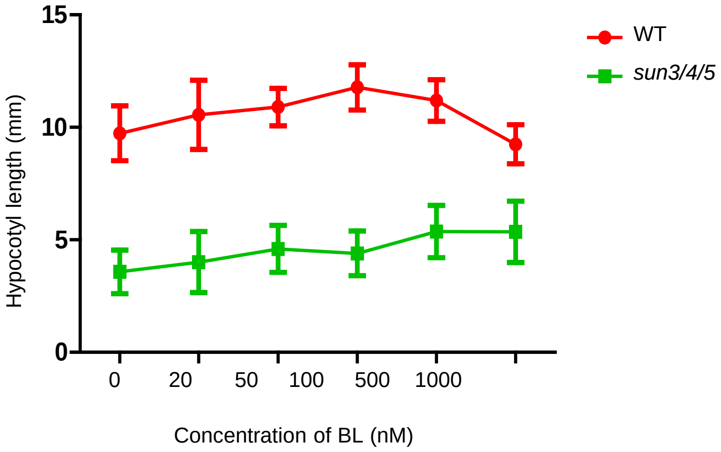

Supplementary Figure 6. **Quantitative analysis of BR sensitivity.**

Hypocotyl length of 5-d-old seedlings grown in the dark on brassinolide (BL)-containing media were measured. Each data point represents the average of 20 seedlings. The values represent means ± s.e.m. 3 independent biological experiments were repeated.

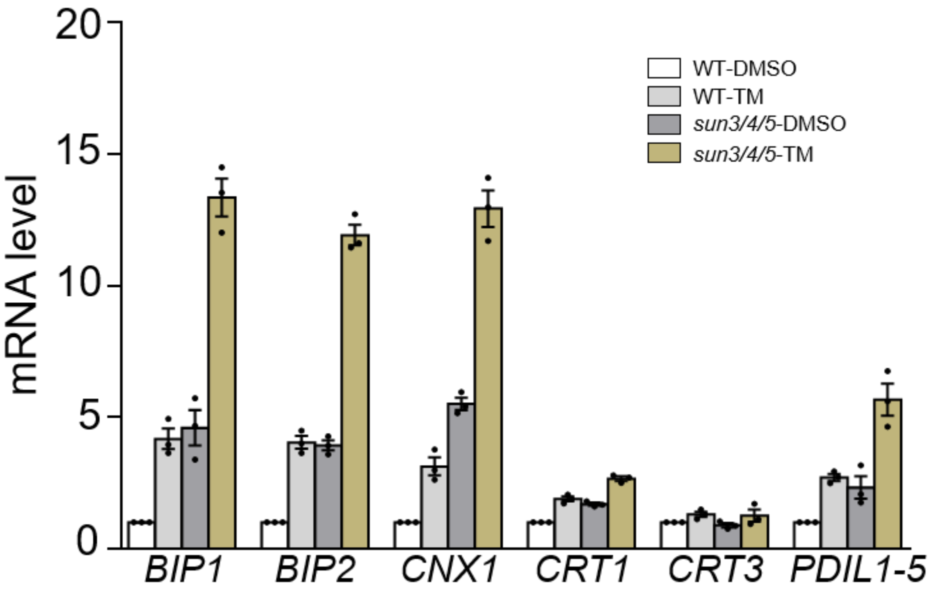

Supplementary Figure 7. The expression level of ER chaperones.

Real-time quantitative RT-PCR analysis of the ER chaperones (*BIP1*, *BIP2*, *CRT1*, *CRT3*, *CNX1* and *PDIL1-5*) in the WT and *sun3/4/5* mutant with and without Tm treatment. The values represent means ± s.e.m. 3 independent experiments were repeated.

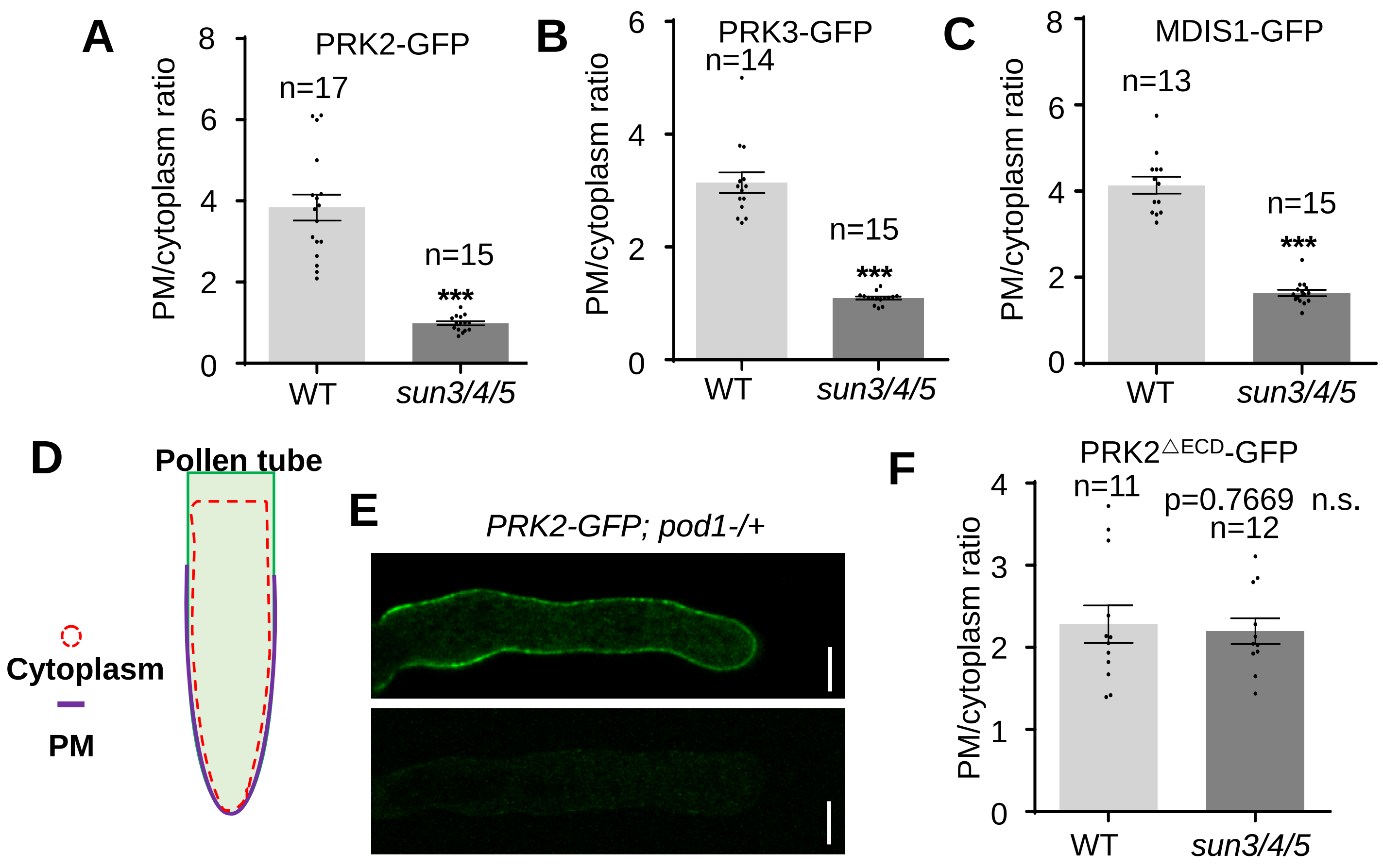

Supplementary Figure 8. Expression of LRR-RLKs in *sun3/4/5* and *pod1/+*.

A. Quantification of the fluorescence intensity ratio of PM/cytoplasm of PRK2-GFP. The values represent means ± s.e.m., Two-tailed Students *t*-test, p*** < 0.001. n = 17 and 15 for WT and *sun3/4/5*, respectively. **B.** Quantification of the fluorescence intensity ratio of PM/cytoplasm of PRK3-GFP. The values represent means ± s.e.m., Two-tailed Students *t*-test, p*** < 0.001. n = 14 and 15 for WT and *sun3/4/5*, respectively. **C**. Quantification of the fluorescence intensity ratio of PM/cytoplasm of MDIS1-GFP. The values represent means ± s.e.m., Two-tailed Students *t*-test, p*** < 0.001. n = 13 and 15 for WT and *sun3/4/5*, respectively. **D**. Schematic diagram of the areas that were used for the measurement of fluorescence intensity. n, number of pollen tubes. dot in the bar graph, the value of each pollen tube. **E**. PRK2-GFP membrane-localization is impaired in 45.7% pollen tubes in *pod1/+*. n = 81 pollen tubes. Bar, 5 μm. **F**. Quantification of the fluorescence intensity ratio of PM/cytoplasm of PRK2$^{\triangle ECD}$-GFP. The values represent means ± s.e.m., Students *t*-test, p = 0.7669. n = 11 and 12 for WT and *sun3/4/5*, respectively.

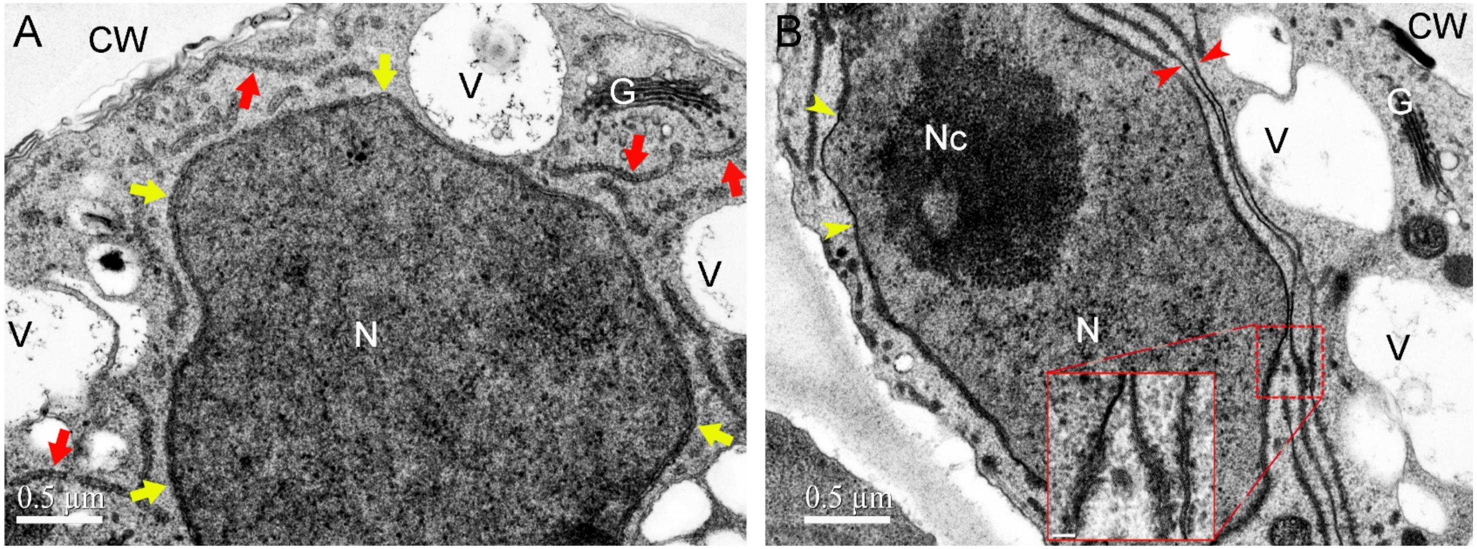

Supplementary Figure 9. TEM images of WT and *sun3/4/5*.

TEM showing the ER, Golgi, nuclei, and vacuoles in the root epidermal cell of the WT (**A**) and *sun3/4/5* (**B**). Red and yellow arrows indicate ER sheets and NE in (**A**), respectively. Arrow heads indicates the narrow ER lumen (**B**). 3 (**A**,**B**) independent biological experiments were repeated. Inset, magnification of the boxed region. V, vacuole; G, Golgi; N, nucleus; CW, cell wall. Bar, 0.5 µm.

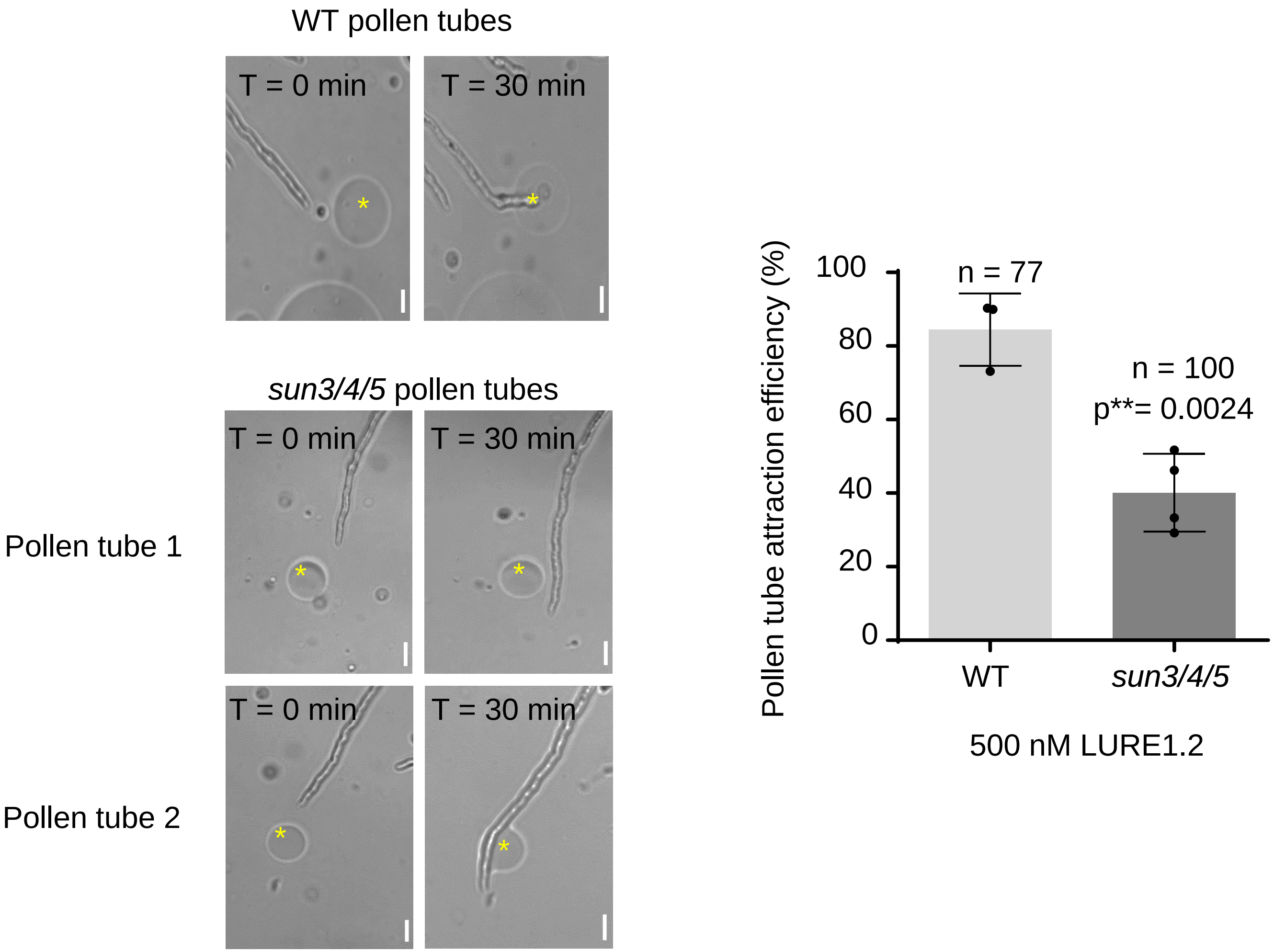

Supplementary Figure 10. Pollen tube attraction efficiency of *sun3/4/5* pollen tubes is reduced.

Statistics of pollen tube attraction efficiency by LURE1.2. The values represent means ± s.e.m., Two-tailed Student's *t*-test, p** = 0.0024. 77 WT and 100 *sun3/4/5* pollen tubes, respectively. Bar = 20 μm.

Supplementary Table 1. Male transmission efficiency of *sun3/4/5* mutants.

Male	×	female	F1 progeny	Progeny	TE
$sun3^{+/-}$ $sun4^{-/-}$ $sun5^{-/-}$		*SUN3 SUN4 SUN5*	*$sun3^{+/-}$ $sun4^{+/-}$ $sun5^{+/-}$*	14	14.4%
			$sun3^{+/+}$ $sun4^{+/-}$ $sun5^{+/-}$	244	
$sun3^{-/-}$ $sun4^{+/-}$ $sun5^{-/-}$		*SUN3 SUN4 SUN5*	*$sun3^{+/-}$ $sun4^{+/-}$ $sun5^{+/-}$*	8	8.9%
			$sun3^{+/-}$ $sun4^{+/+}$ $sun5^{+/-}$	81	
$sun3^{-/-}$ $sun4^{-/-}$ $sun5^{+/-}$		*SUN3 SUN4 SUN5*	*$sun3^{+/-}$ $sun4^{+/-}$ $sun5^{+/-}$*	59	16.1%
			$sun3^{+/-}$ $sun4^{+/-}$ sun5	308	

Supplementary Table 2. Primers used in this study.

Experiment	Name	Sequences (5'-3')
Mutants	LBb1.3	ATTTTGCCGATTTCGGAAC
	SALK_093820-LP	AATCACAACTGCAATAACCGC
	SALK_093820-RP	TCCAATTCTCGAAACAAATCG
	SALK_022028-LP	TTGAACCGGACAAAACTCTTG
	SALK_022028-RP	GGGAATTTCACGGCTTTAAAC
	CS803680-LP	TGACAAATAACCCCACTTTGG
	CS803680-RP	CGAAAGCGATGAAGACAAGAG
	SALK_126070-LP	TAGCAGTATCATGACCCAGCC
	SALK_126070-RP	GTCAGGGAGTCTGAGTTTCCC
RT-PCR	SUN3-RT-F	GTCTCTTTGTCTCTTGTTTTTCTCC
	SUN3-RT-R	AACTGTTTTGTTCATCTGATTTTGC
	SUN4-RT-F	TATGGGGGCTTGTCTTCTTATCTAC
	SUN4-RT-R	TGTATCATTCCCTGGAACTGTGTTA
	SUN5-RT-F	GCGTTCTATCTCTCTCGTTCTCTTA
	SUN5-RT-R	ATCCACAACTTTAGCACCCTTCATA
Tag fusion	PSK-SUN3-GFP-F	ATTCCTGCAGCCGGGGGATCCATGCAAAGGTCGTGTAGA
	PSK-SUN3-GFP-R	GCCCTTGCTCACCATTCTAGAAAGAGACAAAACGAACATA
	PSK-SUN4-GFP-F	ATTCCTGCAGCCGGGGGATCCATGCAAAGATCACGGAGAG
	PSK-SUN4-GFP-R	CCCTTGCTCACCATTCTAGAAAGTGACAAAATGAACATA
	PSK-SUN5-GFP-F	TCGAATTCCTGCAGCCGGGGGATCCATGGCGAGGCGCGGAAGC
	PSK-SUN5-GFP-R	TCGCCCTTGCTCACCATTCTAGAAGAGAGTAAATGAATGAA
	POD1-MYC-F	TTGATATCGAATTCCTGCAGATGGCGATTAGATCCTCG
	POD1-MYC-R	ATGAGCTTTTGCTCGCCCCCGGGATCATTGTGGAGATGGGA
	MYC-CNX1-F	TTCTGAAGAGGACTTGAATGGATCCAGACAACGGCAACTATTT
	MYC-CNX1-R	TGGAGAAACTCGACCGCGGTCTAGACTAATTATCACGTCTCGGT
	SUN3-HA-F	ATAAGCTTGATATCGAATTCCTGCAGATGCAAAGGTCGTGTAGA

	SUN3-HA-R	AACATCATAAGGATAGCCCCCGGGAAGAGACAAAACGAACATA
	POD1-GFP-F	TCGAATTCCTGCAGCCGGGGGATCCATGGCGATTAGATCCTCG
	POD1-GFP-R	TCGCCCTTGCTCACCATTCTAGAATCATTGTGGAGATGGGA
	MYC-CRT3^{N}-F	GGCAAGCTTGGAATTCCTGCAGCC ATGGGATTACCTCAAAATAA
	MYC-CRT3^{N}-R	GCTCTAGAACTAGTGGATCC TCAGTCCCAGTCTGTGTACATAC
	MYC-CRT3^{M}-F	GGCAAGCTTGGAATTCCTGCAGCC ATCCTTCCTCCAAGGAAGAT
	MYC-CRT3^{M}-R	GCTCTAGAACTAGTGGATCC TCAAAGATCTGGATCATCTTCAA
	MYC-CRT3^{C}-F	GGCAAGCTTGGAATTCCTGCAGCCTACGTTCTGAAGTCTATCAA
	MYC-CRT3^{C}-R	GCTCTAGAACTAGTGGATCCTCATAGCTCGTCATGGTAAT
	MYC-CRT3-F	GGCAAGCTTGGAATTCCTGCAGCC ATGGGATTACCTCAAAATAA
	MYC-CRT3-R	GCTCTAGAACTAGTGGATCCTCATAGCTCGTCATGGTAAT
Protein expression in plants	35S:SUN3-GFP-F	CGGGATCCATGCAAAGGTCGTGTAGAACTAGAA
	35S:SUN3-GFP-R	ACGCGTCGACAAGAGACAAAACGAACATAATGAAT
	35S:SUN4-GFP-F	GGGGTACC ATGCAAAGATCACGGAGAGCTCTTC
	35S:SUN4-GFP-R	CGGGATCC AAGTGACAAAATGAACATAACGAAT
	LAT52:SUN4-GFP-F	AACTGCAG ATGCAAAGATCACGGAGAGCTCTTC
	LAT52:SUN4-GFP-R	CGGGATCC AAGTGACAAAATGAACATAACGAAT
	LAT52:PRK2-GFP-F	TAAAAAGCTTGCATGCCTGCAGATGGAATCCAAATGTCTCATGT
	LAT52:PRK2-GFP-R	CTCACCATGGTACCCGGGGATCCTGACAAGTTAATTCCCTCACT
	PRK3:PRK3-GFP-F	CGACGGCCAGTGCCAAGCTTCAGGTTCAGTGCAAGTAGTGT
	PRK3:PRK3-GFP-R	AGAAATAGAACAGCAGTCATGGGTTAAGGAGAAGTAGA
	PRK3:PRK3-GFP-F	TCTACTTCTCCTTAACCCATGACTGCTGTTCTATTTCT
	PRK3:PRK3-GFP-R	ACCATGGTACCCGGGGATCCTCTAGAAAGTGTTACTCGTTCTATCC
	MDIS1:MDIS1-GFP-F	AACGACGGCCAGTGCCAAGCTTAAATATTTAGAACGAATTCC
	MDIS1:MDIS1-GFP-F	GATTCCATCGACAACCCATTTTAGGTATACACCAATGCTAT
	MDIS1:MDIS1-GFP-F	ATAGCATTGGTGTATACCTAAAATGGGTTGTCGATGGAATC
	MDIS1:MDIS1-GFP-R	CCATGGTACCCGGGGATCCTCTAGATGTAGCTTCAGAGGATAAGATC
	LAT52:PRK2$^{\triangle LRR}$-GFP-F	TAAAAAGCTTGCATGCCTGCAGATGGAATCCAAATGTCTCATGT
	LAT52:PRK2$^{\triangle LRR}$-GFP-R	TTCAAACAATTTTGGATCCATACCCATCAACGCCTCGATG
	LAT52:PRK2$^{\triangle LRR}$-GFP-F	CATCGAGGCGTTGATGGGTATGGATCCAAAATTGTTTGAA
	LAT52:PRK2$^{\triangle LRR}$-GFP-R	CTCACCATGGTACCCGGGGATCCTGACAAGTTAATTCCCTCACT
	LAT52:PRK2$^{\triangle ECD-}$GFP-F	TAAAAAGCTTGCATGCCTGCAGATGGAATCCAAATGTCTCATGT
	LAT52:PRK2$^{\triangle ECD}$-GFP-R	CTACAATGTACAAAAACTTGCCATTAACAACCATGAA
	LAT52:PRK2$^{\triangle ECD-}$GFP-F	TTCATGGTTGTTAATGGCAAGTTTTTGTACATTGTAG
	LAT52:PRK2$^{\triangle ECD}$-GFP-R	CTCACCATGGTACCCGGGGATCCTGACAAGTTAATTCCCTCACT
	LAT52:GFP-PRK2^{ECD}-Lit6b-F	CTAGAGGATCCCCGGGTACCAATTCTCTAAGGTCTCTTA
	LAT52:GFP-PRK2^{ECD}-Lit6b-R	CTACGAAAGTGGCTGTACTATTTGGGATTTGACCTGC
	LAT52:GFP-PRK2^{ECD}-Lit6b-F	GCAGGTCAAATCCCAAATAGTACAGCCACTTTCGTAG
	LAT52:GFP-PRK2^{ECD}-Lit6b-R	CAAAAGATGATCTTCAGCGGTACCTCACTTGGTGATGATATAA
	BRI1:BRI1-GFP-F	AACGACGGCCAGTGCCAAGCTTGTTACACTTGCGATAGATAGTTACA
	BRI1:BRI1-GFP-R	CATGGATCCTCTAGAGTCGACTAATTTTCCTTCAGGAACTTCT

	35S:mCherry-HDEL-F	ACTCTAGAGGATCCCCGGGTACCATGGTGAGCAAGGGCGAGGAGGATA
	35S:mCherry-HDEL-R	GAAATTCGAGCTCGGTACCTCATTAGAGCTCATCGTGCTTGTACAGCTCGTCCATGCCGCCG
