# Supplementary material for: POD1-SUN-CRT3 chaperone complex guards the ER sorting of LRR receptor kinases in Arabidopsis
Source: Nat Commun. 2022 May 16;13:2703. doi: 10.1038/s41467-022-30179-w (PMC9110389; doi:10.1038/s41467-022-30179-w)
Supplement: Supplementary file 3 — Description of Additional Supplementary Files [file 41467_2022_30179_MOESM3_ESM.pdf]

Description of Additional Supplementary Files

File Name: Supplementary Data 1
Description: The protein names and sequences used for the phylogenetic analysis.
